# Investigation of brix refractometry for estimating bovine colostrum immunoglobulin G concentration

**DOI:** 10.3389/fvets.2023.1240227

**Published:** 2023-09-25

**Authors:** Donald Sockett, Ryan M. Breuer, Lindsey W. Smith, Nicholas S. Keuler, Thomas Earleywine

**Affiliations:** ^1^Wisconsin Veterinary Diagnostic Laboratory, University of Wisconsin-Madison, Madison, WI, United States; ^2^Department of Medical Sciences, School of Veterinary Medicine, University of Wisconsin-Madison, Madison, WI, United States; ^3^Department of Statistics, University of Wisconsin-Madison, Madison, WI, United States; ^4^Land O'Lakes, Cottage Grove, WI, United States

**Keywords:** colostrum, brix refractometry, immunoglobulin G, transfer of passive immunity, radial immunodiffusion

## Abstract

Many dairy operations uses a Brix refractometer to assess the quality of first-milking colostrum. This study investigated whether a digital Brix refractometer could be used in a model to predict colostrum IgG concentration and whether more than one %Brix threshold could be used for different colostrum IgG concentrations. Colostrum from 182 animals was tested using a digital Brix refractometer and by single radial immunodiffusion. Statistical analysis, using simple linear regression to relate %Brix results with corresponding colostral IgG concentration, and receiver operating characteristic (ROC) analysis were used to identify %Brix cutoffs that had no false positive results. Colostral IgG concentrations from digital Brix refractometry had a *R*^2^ value of 0.818 and a S-value of 21.7 g/L. The large S-value shows that a digital Brix refractometer should not be used in a model to predict colostrum IgG concentration. However, %Brix scores of 19.0, 22.0, 25.0 and 30.0 percent can be used to estimate minimum colostral IgG concentrations of 25, 50, 75 and 100 g/L. These four cutoffs can be used to strategically feed smaller volumes of colostrum to newborn calves. Smaller volumes may reduce unwanted side effects and shorten the time interval in which calves refuse to nurse, while still delivering an adequate mass of IgG to have successful transfer of passive immunity.

## 1. Introduction

Newborn calves are born agammaglobulinemic and are dependent on the successful transfer of passive immunity (TPI) for optimal health and wellbeing (1, 2). Current best practices recommend that calves be fed 150–200 g of bovine colostral immunoglobulin G (IgG) within 2 h of birth, followed by an additional feeding of 75–100 g of IgG, 6–12 h after the first feeding (3). Larger calves (≥35 kg) are typically fed 300 g of IgG, and smaller calves are fed 225 g of IgG (3, 4). Dairy operations testing first-milking colostrum with a Brix refractometer often use a single threshold of ≥22% to ensure that the concentration of IgG is at least 50 g/L (3, 5, 6). The 50 g/L threshold has become the gold standard for first-milking colostrum (2–4). Feeding 4 L of first-milking colostrum within 2 h of birth, followed by an additional 2 L fed 6–12 h later, is a common practice in the industry. Very few calves will voluntarily drink 6 L of colostrum, which necessitates the use of an esophageal tube feeder for delivery (5, 6).

Feeding colostrum using an esophageal tube feeder to a newborn calf is not risk-free. One complication is aspiration pneumonia, which is caused by improper technique or excessive calf movement when the colostrum is delivered. Abdominal distension, colic, and occasionally death are other potential complications of feeding large volumes of colostrum in the first 24 h of life. As such, some dairy calf-care providers and practicing veterinarians have inquired about the possibility of implementing site-specific colostrum management programs. The calf-care providers and veterinarians wish to feed smaller volumes of first milking colostrum but still deliver a sufficient mass of colostral IgG to have excellent TPI. It is hypothesized that feeding smaller quantities of colostrum may improve abomasal health, reducing the likelihood of abdominal distension and colic post-colostrum feeding. The aim of this study was to compare %Brix results to the IgG concentrations of bovine colostrum at first milking and determine four different %Brix thresholds for 25, 50, 75, and 100 g/L of bovine colostral IgG.

## 2. Materials and methods

### 2.1. Colostrum sample collection and testing

Four Holstein dairy farms located in the Midwestern region of the United States submitted first-milking colostrum samples for testing and analysis at an accredited American Association of Veterinary Laboratory Diagnosticians (AAVLD) laboratory, the Wisconsin Veterinary Diagnostic Laboratory (WVDL). Power calculations based on the correlation between the Brix score and IgG concentration using the Power procedure in SAS (SAS 9.4, SAS Institute Inc., Cary, NC, USA) were used to calculate the sample size needed for each of the five different sampling strata. With an alpha of 0.05 and a power of 0.80, the projected minimum sample size for each of the five strata was 7 when the correlation was 0.65 or higher. Sampling kits were sent to each farm with instructions on how many samples should be collected from each of the five different sampling strata (Table 1). One hundred and eighty-two first-milking colostrum samples were collected from multi- and primiparous Holstein dairy cows. Colostrum samples were tested immediately after harvesting on-farm with a temperature-compensating digital Brix refractometer (Misco, Solon, Ohio) to obtain a %Brix reading. The refractometer was calibrated using distilled water at least once a week. If the samples met the predetermined sampling criteria, the %Brix result was recorded, and ~25–35 ml of colostrum were collected into 50 ml conical centrifuge tubes as a combined quarter sample and frozen immediately after collection at −20°C until shipped to the WVDL for testing and analysis. Samples were shipped with a sufficient number of cold packs to ensure the samples remained frozen. After receipt, each colostrum sample was assigned a unique bar-coded identification number and immediately frozen at −80°C.

**Table 1:** Stratified sample design.

**Strata number**	**%Brix**	**Sample number**
1	< 18.0	22
2	18.0–21.9	43
3	22.0–26.9	56
4	27.0–31.9	45
5	≥32.0	16
Total		182

Frozen (−80°C) colostrum samples were thawed in a bead bath. Testing commenced when the colostrum samples reached a temperature of 38.5°C to mimic the average normal bovine body temperature. The samples were thoroughly vortexed and tested in duplicate using a digital Brix refractometer, the same instrument model used in the on-farm collections. The Brix refractometer was calibrated by laboratory personnel using deionized water before testing commenced and repeated after 10 colostrum samples were tested. The samples were tested for IgG concentration using a commercially available single radial immunodiffusion (SRID) test (#72841, Triple-J Farms, Bellingham, WA) using the same methodology as previously described (7). Each SRID plate was set up with two internally developed bovine serum controls (high- and low-IgG concentration) in addition to the three standard solutions provided by the manufacturer. The samples were tested in duplicate. SRID agar plates were placed in a moist chamber and incubated at 23.5°C for 24 h. After incubation, the precipitin ring diameter for each sample, including the controls, was measured using a digital electronic caliper with a resolution of 0.01 mm. Samples with a coefficient of variation (CV) >5% were retested. Any colostrum sample in which the farm %Brix result differed by more than 0.5 %Brix from the WVDL was retested a second time to verify the results.

### 2.2. Statistical analysis

The %Brix results were compared with their matching SRID results using simple linear regression (GraphPad Prism ver 9.5, La Jolla, California). The %Brix was the independent variable, and the colostral IgG concentration, measured by SRID, was the dependent variable. The regression model was used to predict colostral IgG concentration from each of the 182 %Brix results. As previously described, the model was also used to calculate residual values for each of the 182 colostrum samples (8). All SRID results with a residual IgG value ± 10 g/L or higher were retested in duplicate. The coefficient of determination (*R*^2^) and standard error of the regression (S) were calculated, and a residual plot was constructed. Receiver operating characteristic (ROC) analysis using the ROCR package (R Foundation for Statistical Computing, ver. 4.04, Vienna, Austria) was used to calculate the cutoffs for colostral IgG concentrations of 25, 50, 75, and 100 g/L such that there were no false positive (FP) results for each %Brix value and the corresponding IgG result. An FP was defined as a %Brix value that caused the model to overestimate the true colostral IgG concentration.

## 3. Results

### 3.1. %Brix and colostrum SRID analysis

**Figure 1:**
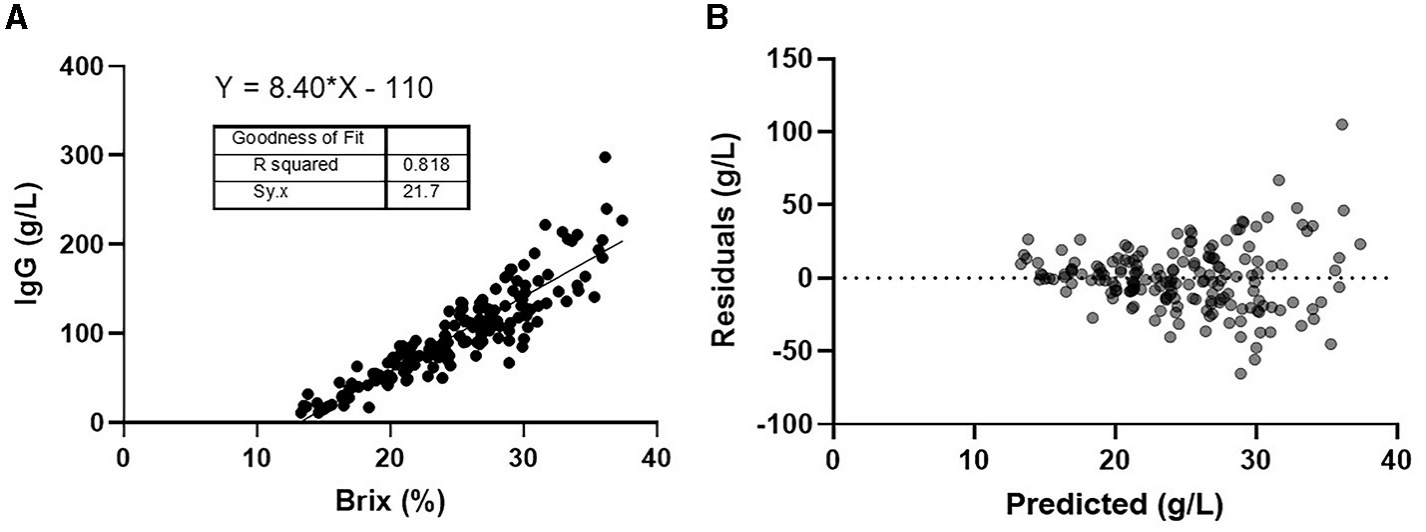
**(A)** Linear regression of Brix (%) vs. IgG g/L. **(B)** Residual plot of predicted colostral IgG concentration vs. actual concentration.

The linear regression model was estimated to be y = 8.40·X −110, where X was the %Brix score, and Y was the colostral IgG concentration. The *R*^2^ value for comparing %Brix and the corresponding colostral IgG concentration was 0.818 with an S-value of 21.7 g/L (Figure 1A). A graphical depiction of the residual plot (Figure 1B) shows the difference between the model's predicted IgG concentration (horizontal line) based on the %Brix score and the colostral IgG concentration when measured by SRID. The Brix cutoffs for estimating colostral IgG concentrations of 25, 50, 75, and 100 g/L were determined to be 18.7, 21.4, 24.7, and 30.1%, respectively. We propose using rounded values of 19.0, 22.0, 25.0, and 30.0 for simplicity (Table 2).

**Table 2:** Actual and proposed %Brix thresholds for 25, 50, 75 and 100 g/L.

**Colostrum IgG g/L**	**Calculated %Brix**	**Proposed %Brix**
≥25	18.7	19.0
≥50	21.4	22.0
≥75	24.7	25.0
≥100	30.1	30.0

## 4. Discussion

This study shows that digital Brix refractometry cannot accurately estimate first-milking colostrum IgG concentration. However, it can be used to estimate the minimum amount of IgG for predetermined %Brix cutoffs of first-milking colostrum concentrations of 25, 50, 75, and 100 g/L. There is high confidence in the feasibility of the proposed %Brix cutoffs for on-farm use because the digital Brix refractometer has high precision and produces very reproducible results in the field (9–12). An examination of Figures 1A, B shows that as %Brix scores increase, the digital refractometer does not measure as well, particularly when the Brix scores are 30% or higher. This observation is likely caused by increasing concentrations of fat, casein, and other macromolecules that are insoluble in water, thus impacting the Brix refractometer reading (13).

A stratified sampling method was used to collect colostrum samples following screening with a digital Brix refractometer (14). This design differs from previous reports that collected colostrum samples without prior Brix refractometer screening (9–12). Stratified sampling has the advantage of ensuring a greater high to low range of %Brix scores naturally occurring in dairy herds, reducing testing costs. There were also stringent laboratory quality control measures employed that were not mentioned in previous reports (9–12). Bovine colostrum has a higher viscosity than milk (6), which makes accurate pipetting of small volumes especially difficult when the samples are cold/chilled to below body temperatures. This is the reason colostrum samples were thawed and warmed to 38.5°C (normal body temperature of cattle) and thoroughly mixed prior to pipetting. The commercial SRID test specifies that 5 μL of sample be pipetted into each well of an agar plate. Calibrated pipettes that are checked and verified semi-annually were used in the study because inaccurate pipetting of small volumes of analyte can greatly affect the accuracy of test results (15).

The *R*^2^ value of 0.818 suggests a strong relationship between %Brix and colostral IgG concentration. However, many reports have not calculated S-values or constructed a residual plot (9–12). When critically examined, this additional analysis causes a distinctly different picture to emerge for the usefulness of the %Brix score in predicting colostral IgG concentration. The S-value is an estimate of the average distance that the true measured colostrum IgG concentration falls from the concentration predicted by the model (8). For our model, S = 21.7 g/L. Thus, an approximate 95% prediction interval for a single new colostrum sample is ±1.96^*^S, for an estimate ±42.5 g/L. For example, if a %Brix value of 22% is used, the linear model predicts a colostral IgG concentration of 74.8 g/L, but the true value is only suspected to lie somewhere between 32.3 and 117.3 g/L with 95% confidence. This problem is illustrated graphically with the residual plot in Figure 1B. A prediction accuracy of ±42.5 g/L leads to the conclusion that model prediction of colostral IgG concentration based on %Brix score does not provide sufficient accuracy to be useful in predicting actual IgG concentration.

Several studies have shown both the short-term and long-term benefits of having adequate TPI in calves (16–19). It is well-established that calf health and wellbeing, future milk production, and longevity in the herd are highly correlated with successful TPI. For these reasons, using a model that often overestimates bovine IgG concentration based on %Brix scores when a fixed volume of colostrum is fed to newborn calves is unacceptable. The thresholds for the colostral IgG concentration of 25, 50, 75, and 100 g/L do not provide an estimate of the IgG concentration in the colostrum; it only informs the operator of the minimum amount of IgG that is likely to be present for each of the four different cutoffs. For example, a %Brix score of 30.0 only indicates that it is very likely that there is at least 100 g/L of bovine IgG in the colostrum sample. The Brix refractometer should only be used to estimate the minimum amount of IgG in a colostrum sample for the four different cutoffs and not be used in a model to predict individual colostral IgG concentrations.

This study was requested by some dairy calf-care providers and veterinarians due to their unhappiness with current first-milking colostrum feeding guidelines. On-farm observations by calf-care providers of calves with abdominal distension causing discomfort and/or colic, when coupled with the fact that newborn calves often refuse to nurse for an extended period of time, post-colostrum feeding, has caused concerns for calf health and wellbeing. A previous study has shown that first-milking-colostrum feedings can form a large curd that may be retained in the abomasum for more than 8 h after ingestion (20). It has also been reported that the volume of colostrum fed to newborn calves had less influence on the efficiency of immunoglobulin absorption than did the colostral IgG concentration itself (21). Calves fed 1 L of high-quality colostrum had more efficient immunoglobulin absorption than calves fed the same mass of immunoglobulin in 2 L of colostrum (21). Based on these observations and the results of this study, calf-care providers and veterinarians should consider feeding newborn calves a smaller volume of first milking colostrum (2–3 L) that contains at least 75–100 g/L of IgG. The first feeding should be followed by a second feeding of 1.0–1.5 liters of colostrum that contains at least 75–100 g/L of IgG. Colostrum that contains <75–100 g/L of bovine IgG can be fortified with a high-quality colostrum replacement product (22, 23). The concept is to feed a smaller but more concentrated volume of colostrum to newborn calves that delivers an adequate mass of immunoglobulins to have successful TPI. Feeding a smaller colostrum volume will reduce the number of newborn calves that refuse to nurse for at least 18–36 h post-colostrum feeding. Smaller colostrum volumes may also reduce the incidence of aspiration pneumonia, improve forestomach and abomasal health, and perhaps eliminate colic caused by over-distension of the abomasum with the formation of a large colostrum curd (20). Finally, it will increase the farm supply of colostrum that can be used to fortify newborn calf liquid feed diets for the first 2–14 days of life. Daily feeding of small volumes of first-milking colostrum for the first 2–14 days of a calf's life has improved gut health, increased average daily gain, and reduced neonatal calf diarrhea and pneumonia (24, 25).

## 5. Conclusion

This study shows that models that use digital Brix refractometry cannot accurately predict first-milking colostrum IgG concentration. Knowing this, calf-care providers and veterinarians should be aware that they should only use %Brix predetermined cutoffs when managing colostrum feeding programs. First-milking colostrum with higher %Brix scores (≥25%) when used to feed smaller volumes of colostrum to newborn calves is an intriguing concept that warrants further study.

## Data availability statement

The original contributions presented in the study are included in the article/supplementary material, further inquiries can be directed to the corresponding author.

## Ethics statement

Ethical review and approval was not required for the study on animals in accordance with the local legislation and institutional requirements.

## Author contributions

DS: study design, data analysis, testing, writing—original draft, and editing. RB: writing—review, editing, and assistance with document submission. LS: laboratory testing supervision, writing—review, and editing. NK: statistical and data analysis, writing—review, and editing. TE: study design, writing—review, and editing. All authors contributed to the article and approved the submitted version.
